# The Global Diversity of Sea Pens (Cnidaria: Octocorallia: Pennatulacea)

**DOI:** 10.1371/journal.pone.0022747

**Published:** 2011-07-29

**Authors:** Gary C. Williams

**Affiliations:** California Academy of Sciences, San Francisco, California, United States of America; National Institute of Water & Atmospheric Research, New Zealand

## Abstract

Recent advances in deep-sea exploration technology coupled with an increase in worldwide biotic surveys, biological research, and underwater photography in shallow water marine regions such as coral reefs, has allowed for a relatively rapid expansion of our knowledge in the global diversity of many groups of marine organisms. This paper is part of the PLoS ONE review collection of WoRMS (the Worldwide Register of Marine Species), on the global diversity of marine species, and treats the pennatulacean octocorals, a group of cnidarians commonly referred to as sea pens or sea feathers. This also includes sea pansies, some sea whips, and various vermiform taxa. Pennatulaceans are a morphologically diverse group with an estimated 200 or more valid species, displaying worldwide geographic and bathymetric distributions from polar seas to the equatorial tropics and from intertidal flats to over 6100 m in depth. The paper treats new discoveries and taxa new to science, and provides greater resolution in geographic and bathymetric distributions data than was previously known, as well as descriptions of life appearances in life and *in situ* observations at diverse depth.

## Introduction

### Human Interests

Certainly one of the earliest accounts of pennatulacean natural history is from the anonymous chronicler of Captain James Lancaster’s voyage to the East Indies in 1601 [1]. The following description probably refers to a species of *Virgularia* that inhabits the intertidal sand flats of islands in the Sombrero Channel of the Nicobar Islands. One species, *Virgularia juncea* (Pallas, 1766), is known from the eastern Indian Ocean and Australian region as well as the Andaman Islands. The narrator states, “Upon the sands of this island of Sombrero we found a small twig growing up like a young tree, and on offering to pluck it up, it shrinks down to the ground, and sinks, unless held very hard. On being plucked up, a great worm is found to be its root, and as the tree growth in greatness, so doth the worm diminish; and as soon as the worm is entirely turned into tree, it rooteth in the earth, and so becomes great. This transformation is one of the strangest wonders that I saw in all my travels: For, if this tree is plucked up when young, and the leaves and bark stripped off, it becomes a hard stone when dry, much like white coral: thus is this worm twice transformed into different natures. Of these we gathered and brought home many.” In affirmation of the first part of the above quoted passage, Charles Darwin describes the rather striking ability of a particular species of sea pen (in the genus *Virgularia* from an intertidal area in Patagonia) to quickly and forcefully withdraw itself into the sandy mud when disturbed [2].

Several historical passages worthy of note regarding bioluminescence are from oceanographic explorations of the latter half of the nineteenth century. For example, Wyville Thomson of the *HMS Challenger* expedition, reports, “Many of the animals were most brilliantly phosphorescent …. In some place nearly everything brought up seemed full of luminous sparks. The alcyonarians, the brittle-stars, and some annelids were the most brilliant. The *Pennatulae*, the *Virgulariae*, and the *Gorgoniae* shone with a lambient white light, so bright that it showed quite distinctly the hour on a watch …. We had another gorgeous display of luminosity during this cruise …. The dredge came up tangle with the long pink stems of the singular sea-pen *Pavonaria quadrangularis*. The *Pavonariae* were resplendent with a pale lilac phosphorescence like the flame of cyanogen gas: not scintillating …., almost constant, sometimes flashing out at one point more brightly and then dying gradually into comparative dimness, but always sufficiently bright to make every portion of a stem caught in the tangles or sticking to the ropes distinctly visible. From the number of specimens of *Pavonaria* brought up at one haul we had evidently passed over a forest of them. The stems were a metre long, fringed with hundreds of polyps [3].” Likewise, Tizard states, “Many of the Pennatulida are known to be phosphorescent, and in this specimen of *Umbellula*, when taken from the trawl, the polyps and the membrane covering the axis of the stem exhibited a most brilliant phosphorescence. A like phenomenon was observed in the case of many other Alcyonarians obtained from the deep sea,... *Umbellula* was long one of the rarest of zoological curiosities. The first specimens ever described were obtained on the coast of Greenland, early in the last century, by Captain Adriaanz, commander of the 'Britannia,' while on a whale-fishing expedition; on this occasion two specimens were found adhering to the sounding line at a depth of 236 fathoms. These were described by M. Christlob Mylius, and one of them was again described in the Philosophical Transactions for 1754, in a letter from Mr. John Ellis to Mr. Peter Collinson, 'Concerning a cluster-polyp found in the sea near the coast of Greenland.' Mr. Ellis compared it to the '*Encrinos* or *Lilium lapideum*...', and indeed the resemblance to a Crinoid is not a little striking. For more than a century the animal was not seen again, and it is only a few years since two specimens were dredged in deep water during the cruise of the Swedish ships 'Ingegerd' and 'Gladan', in the Arctic Ocean. These were described in 1874 by J. Lindahl as two new species, --- *Umbellula minacea* and *Umbellula pallida* [4].”

## Methods

Apart from a variety of sources gleaned from a rich literature on the Pennatulacea dating back to the Fifteenth Century, observations on living pennatulaceans have been conducted by the author over the past three decades. Field observations and collections of these organisms have been made in California, South Africa, Palau, Bali, Papua New Guinea, the Solomon Islands, and the Philippines. The methods that have been employed include SCUBA diving, deep-water dredging and trawling, and the use of ROV’s (Remotely Operated Vehicles).

## Results

### Anatomy and Morphology

**Figure 1:**
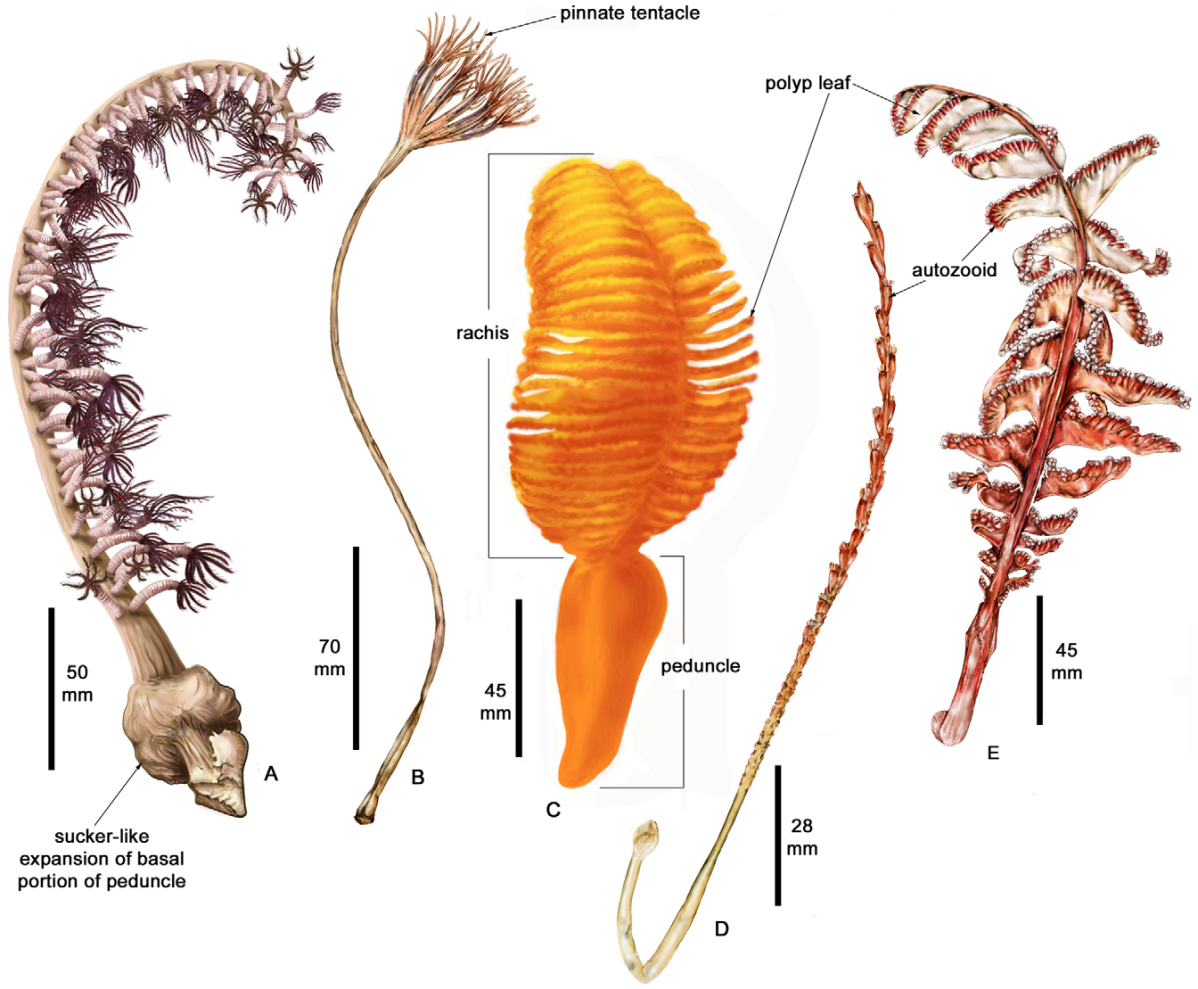
A. *Anthoptilum* sp. B. *Umbellula lindahli*. C. *Ptilosarcus gurneyi*. D. *Protoptilum carpenteri*. E. *Pennatula* sp. Illustrations by Laura Garrison (C), Stephanie King (A), and Jessica Machnicki (B, D, E), California Academy of Sciences.

Pennatulaceans are a specialized and morphologically distinct group of octocorallian cnidarians [5]. Unlike other octocorals, they are formed by a single large primary polyp, also known as the initial polyp or oozooid. The primary polyp is anchored into soft sediment by a proximal muscular peduncle. The distal region of the primary polyp forms a rachis that contains a few or many secondary or daughter polyps, which arise laterally from the central unbranched rachis as autozooids (the larger feeding polyps) and siphonozooids (smaller polyps for water circulation). Some species have conspicuous polyp leaves, often strap-shaped wing-like expansions, which can contain numerous autozooids (Fig. 1C, E and [6]–[8]). Some species, such as *Pennatula inflata*, may also have polyps intermediate in form known as mesozooids. The common name “sea pen” and the group name “Pennatulacea” are from the resemblance of the species in some genera (such as *Pennatula*, *Pteroeides*, and *Virgularia*) to quill pens resulting from the possession of conspicuous polyp leaves. Pennatulacean sclerites (spicules), do not for the most part exhibit a great diversity of shapes and ornamentation, as in other octocorals, but are usually smooth three-flanged spindles, sometimes ovals, or rarely irregularly-shaped plates. The thin tissue of sea pens is called the coenenchyme, and as in all octocorals, is composed of three layers: the outer epidermis and an inner gastrodermis, with a mesogleal layer in the middle. The mesoglea is where the sclerites are formed by specialized amoeboid cells known as scleroblasts. Internally, most pennatulaceans have a central axis composed primarily of calcium carbonate, which runs partially or entirely along the length of the animal, and is circular or quadrangular in cross-section. Some pennatulaceans do not have axes, such as in the genera *Renilla*, *Echinoptilum*, *Actinoptilum*, as well as some veretillids.

### Ecology

**Figure 2:**
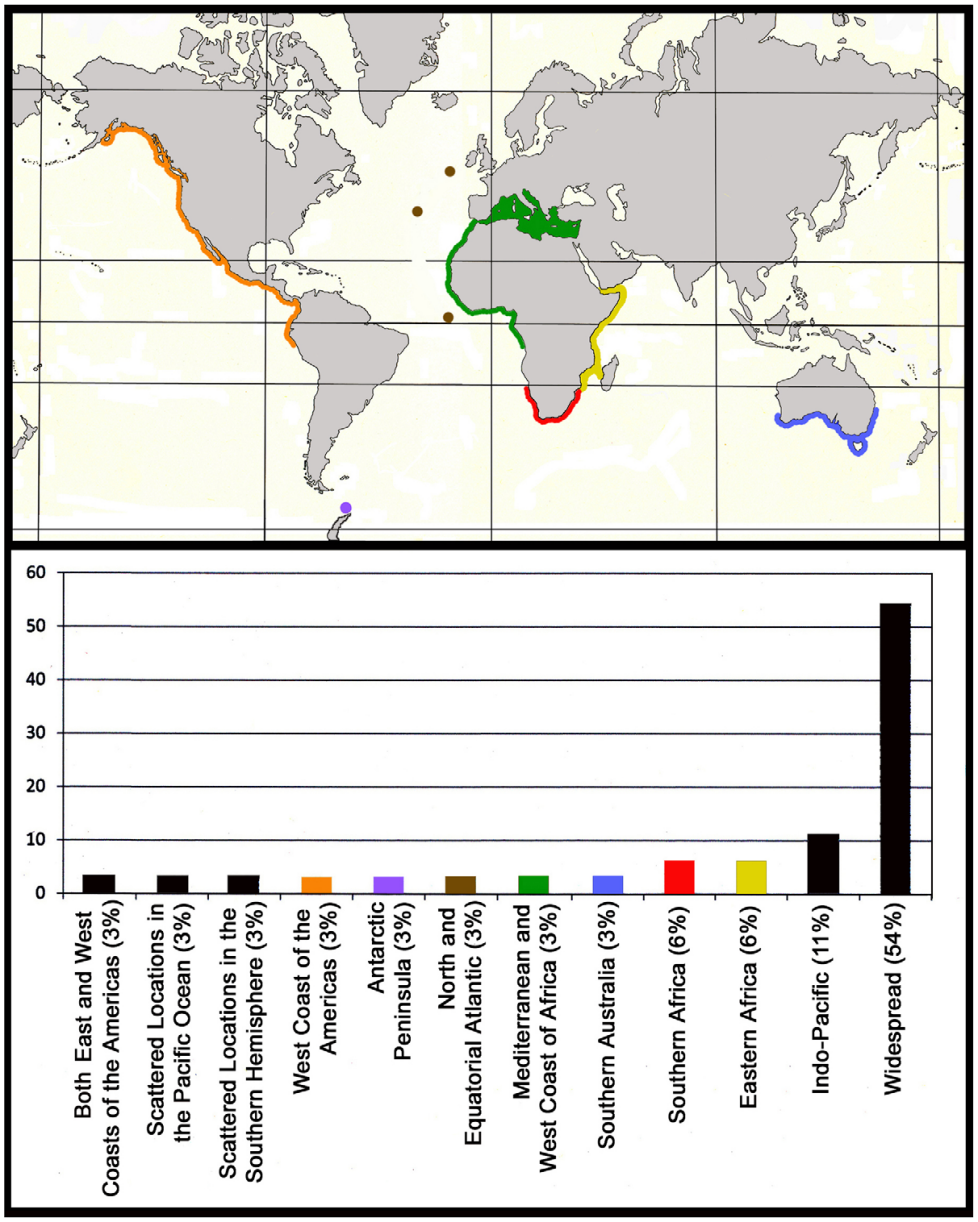
Biogeographic distribution of pennatulacean genera with geographically restricted ranges. Colors represent specific endemic regions for particular genera: *Ptilosarcus* (orange); *Gilibelemnon* (violet); *Porcupinella* (brown); *Sarcoptilus* (blue); *Crassophyllum* (green); *Amphibelemnon* and *Actinoptilum* (red); *Scytaliopsis* and *Amphiacme* (yellow). Black bars represent widespread regions. Vertical axis represents percentage of total number of pennatulacean genera (n = 35). Sources: [7], [40]–[43], [45].

**Figure 3:**
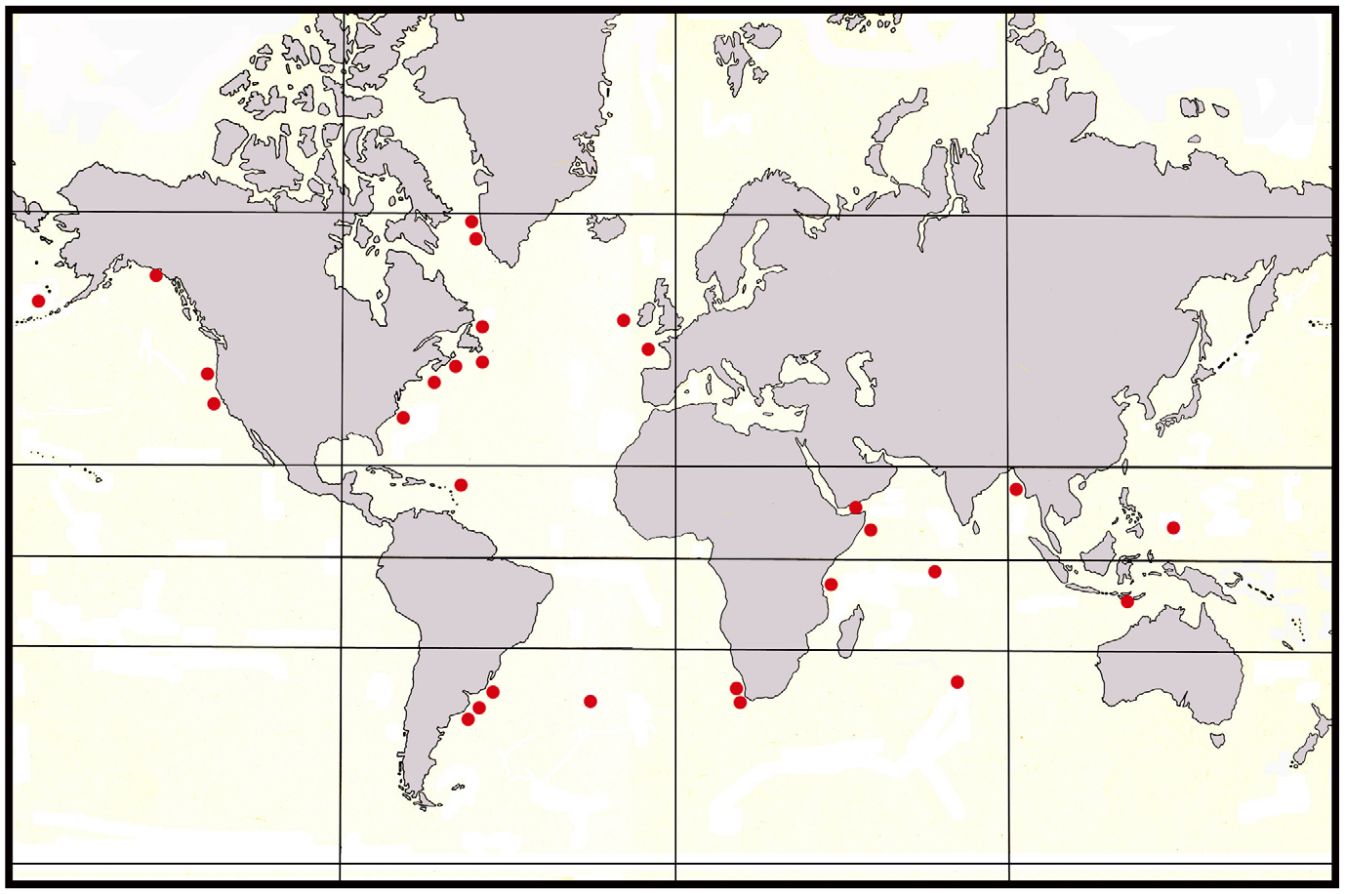
Widespread distribution of the pennatulacean *Anthoptilum grandiflorum* (• =  collecting stations). Sources: Acuña & Zamponi, 1992; Grasshoff, 1982; Hickson, 1916; Kolliker, 1880; Kukenthal & Broch, 1911; Williams, 1992. Sources: [11], [19], [23], [26], [37]–[38].

**Figure 4:**
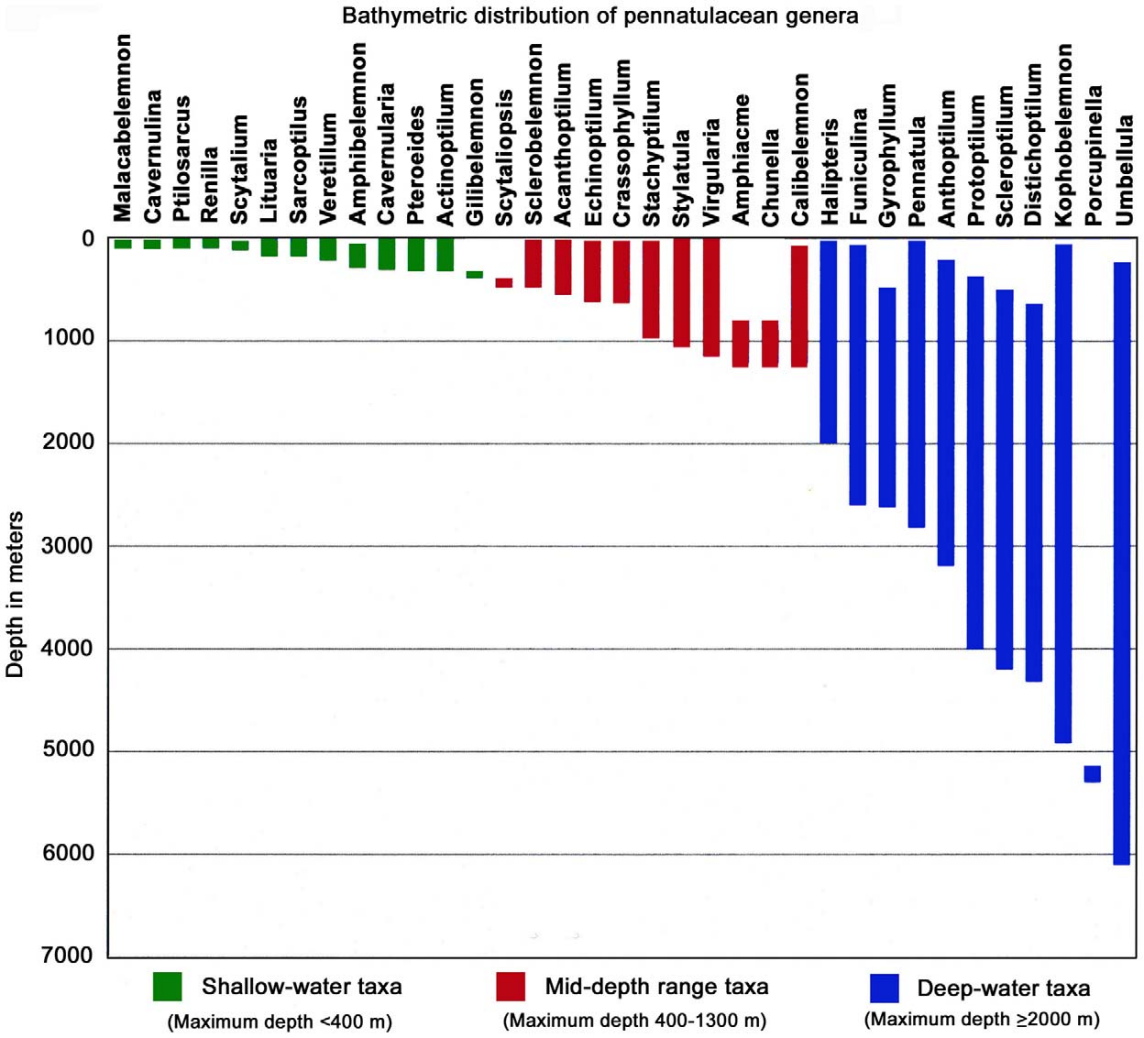
Bathymetric distributions of pennatulacean genera. Sources: [11], [16], [40]–[41], [45]. The demarcation between bathymetric groups is arbitrary: shallow <400 m, mid-range 400-13—m, deep ≥2000 m.

Pennatulaceans have been encountered in all the world’s oceans, from polar to equatorial latitudes, and from intertidal regions to at least 6100 m in depth (Table 1, Figs. 2, 3, 4). A few species such as *Anthoptilum* sp. are even capable of inhabiting rocky surfaces by a sucker-like expansion of the proximal end of the peduncle (Fig. 1A). Many benthic marine environments have been colonized by pennatulaceans. Included here are intertidal sand or mud flats, sandy areas on or near coral reefs, shallow-water areas of mud or rubble, continental shelves and slopes, rocky outcrops between approximately 600 and 2000 m in depth, abyssal plains, and hadal trenches. Most species have a muscular peduncle for anchoring in soft sediments, and thus are able to inhabit vast areas of relatively uniform benthic environments such abyssal plains. Consequently, several species have extremely wide distribution, virtually circum-global in some cases. Dispersal potential is undoubtedly of fundamental importance regarding patterns of restricted vs. widespread geographic ranges. It is postulated that some octocorals that are restricted endemics or exhibit relatively narrow ranges, brood embryos internally and the larva either crawl a short distance from the adult or are short-lived in the plankton and subsequently establish new colonies in near proximity to the adults. Contrasted with this, species that have long-lived planktonic larvae may travel long distances in prevailing currents, thus giving rise to widespread geographic ranges.

**Table 1:** The thirty-five extant pennatulacean genera – estimated numbers of valid species and geographic distribution; from various sources [7], [19], [47].

Genus	Spp. No.	Distribution	Genus	Spp. No.	Distribution
*Acanthoptilum*	8	North America & New Zealand	*Malacobelemnon*	3	Antarctic, southeastern Africa, eastern Australia
*Actinoptilum*	1	South Africa	*Pennatula*	14	Worldwide Distribution
*Amphiacme*	1	Western Indian Ocean	*Porcupinella*	1	North Atlantic
*Amphibelemnon*	1	Southwestern Africa	*Protoptilum*	6	Atlantic & Pacific Oceans
*Anthoptilum*	5	Circumglobal	*Pteroeides*	25+	Western Pacific & Eastern Atlantic
*Calibelemnon*	3	Western Atlantic & Indo-Pacific	*Ptilosarcus*	2	Eastern Pacific
*Cavernularia*	14	Eastern Atlantic & Indo-Pacific	*Renilla*	6	North & South America
*Cavernulina*	4	Pacific & Indian Oceans	*Sarcoptilus*	4	Southern Australia
*Chunella*	1–3	Indo-West Pacific	*Sclerobelemnon*	8	Western Atlantic & Indo-West Pacific
*Crassophyllum*	2	Eastern Atlantic & Mediterranean	*Scleroptilum*	1	Scattered in Atlantic, Indian & Pacific Oceans
*Distichoptilum*	1	Circumglobal	*Scytaliopsis*	1	Western Indian Ocean
*Echinoptilum*	6	Indo-Pacific	*Scytalium*	3	Indo-West Pacific
*Funiculina*	3	Circumglobal	*Stachyptilum*	3	Pacific Ocean
*Gilibelemnon*	1	Antarctic Peninsula	*Stylatula*	10	North Atlantic, Eastern Pacific, New Zealand
*Gyrophyllum*	2	North Atlantic, Indian Ocean, Western Pacific	*Umbellula*	9	Worldwide Distribution
*Halipteris*	6	Circumglobal	*Veretillum*	7	Eastern Atlantic & Indo-West Pacific
*Kophobelemnon*	9	Circumglobal	*Virgularia*	20	Circumglobal
*Lituaria*	10	Indo-West Pacific			

Deeps-sea pennatulaceans are often found in relatively eutrophic conditions in moderately high energy environments and consequently may exhibit patchy distributions [9]. An appreciable bottom current is necessary for procuring sufficient food and hence deep-sea pennatulaceans are often most common in more active areas such as sea mounts, escarpments, continental slopes, and along the bases of ridges [10]–[11].

Due to the ability of pennatulaceans to exploit marine benthic habitats comprised of unconsolidated sediments such as mud, sand, fine rubble, or abyssal ooze, many species are able to inhabit extensive regions of the sea bottom, unlike many other sedentary organisms that need suitable hard substrata for settlement and attachment [11]. At least 71% of pennatulaceans at the familial level and approximately 54% at the generic level have extremely widespread geographic distributions, several of these show virtually cosmopolitan ranges. The remaining 46% of the genera are geographically restricted to various regions of the world’s oceans (Fig. 2). Several species in particular exhibit widespread to nearly worldwide distributions, including *Anthoptilum grandiflorum* (Fig. 3), *Distichoptilum gracile*, *Funiculina quadrangularis*, and *Umbellula lindahli*. Shallow water species such as *Actinoptilum molle*, *Sarcoptilus grandis*, and *Ptilosarcus gurneyi*, are endemic to isolated geographic regions – southern Africa, southern Australia, and the Pacific coast of North America, respectively (Table 1 and [7]). Shallow-water genera living in areas of soft sediment associated with coral reefs include *Cavernularia*, *Veretillum*, *Sclerobelemnon*, *Scytalium*, *Virgularia* and *Pteroeides* [12].

The thirty-five extant pennatulacean genera can be divided into three groups based on their bathymetric ranges: shallow-water (0–400 m), mid-depth range (400–2000 m), and deep-water (2000–6100 m). Species with greater depth ranges often tend to have greater geographic ranges. This is to be expected with the deep-sea being more homogenous than coastal environments. The three deepest known pennatulaceans belong to the genera *Kophobelemnon*, the recently described *Porcupinella* from the Atlantic Ocean, and *Umbellula* (Fig. 4).

Williams (1999: 49–55) provides extensive bibliographic sources regarding pennatulacean behavior, bioluminescence, physiology and cell biology, and natural products chemistry including biochemistry, and toxicology [8]. Physiological studies include investigations of daily rhythmic activities of *Cavernularia*, neural physiology in *Veretillum* and *Cavernularia,* behavioral biology in *Renilla*, and bioluminescence in a variety of pennatulacean taxa. Bioluminescence has been recorded in at least twenty one species of pennatulaceans and many more are likely to have such capabilities [13]. Bioluminescence in sea pens is most likely for defense, to startle and ward off potential predators. Some virgulariids are capable of rapidly disappearing into the sediment when disturbed (see Introduction – Human Interests). At least two shallow-water species (in the genera *Cavernularia* and *Virgularia*) harbor zooxanthellae and are active diurnally. Other shallow-water species are nocturnal and withdraw into the surrounding sediment during the day, emerging at night to feed.

### History of Discovery

The common name “sea pen” comes to us from the ancient Romans who had knowledge of pennatulaceans and called them “Penna marina” (sea feathers or sea pens), or referred to them as “Mentula alata” (winged penis). They apparently made no record of their ability to exhibit luminescence, as most published accounts of pennatulaceans prior to 1758 treated observations on the light producing capabilities of sea pens. The first published record of bioluminescence in sea pens is by Conrad Gesner in his 1555 work, “De lunariis” [14]. Bioluminescence in sea pens is at least briefly mentioned between 1558 and 1746 by François Boussuet, Caspar Bauhin, Ulisse Aldrovandi, Johann Bauhin, and Thomas Shaw [8].

Early accounts between 1469 and 1601, treating the natural history of octocorals in general (including pennatulaceans) include Pliny the Elder, Guillaume Rondelet, Conrad Gesner, Ferrante Imperato, and James Lancaster. In addition, some other noteworthy pre-Linnean accounts dealing at least partially with octocorals, include those of Francisci Erasmi, Paolo Boccone, Hans Sloane, Jacques Barrelier, Herman Boerhaave, Christlob Mylius, and John Ellis [8]. Rondelet and Gesner recognized the animal nature of many marine organisms (including some coelenterates), which were previously viewed as transitional between plants and animals (the zoöphytes of the Roman scholars Sextus Empiricus and Pliny the Elder). The early perception of corals and other organisms as zoöphytes was based on Aristotle’s concept of the gradation from plants to animals. This served to promote a state of misconception for roughly two thousand years until 1753 when Jean-André Peyssonnel [8], through his observations of the movement and function of coral polyp tentacles, firmly established corals as animals and their inclusion with other coelenterates.

**Figure 5:**
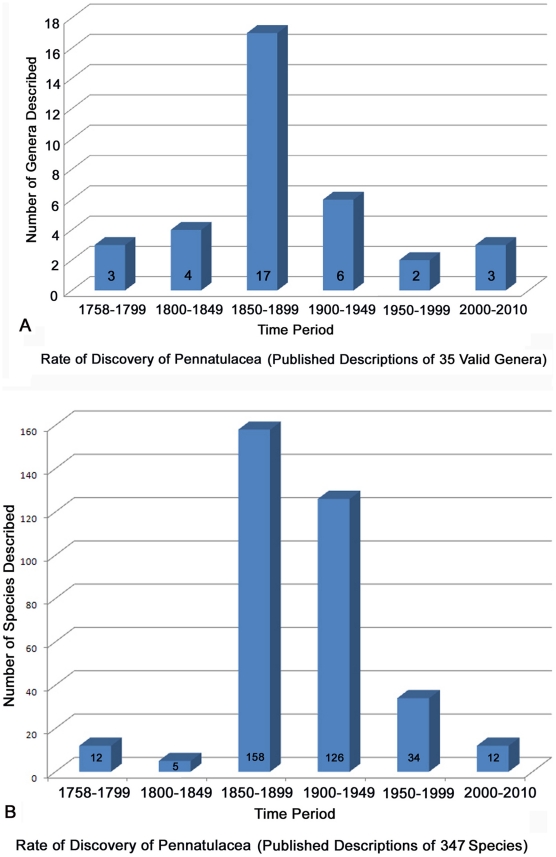
Rate of discovery of Pennatulacea. A. Number of published descriptions of valid genera from 1758 to present. B. Number of published descriptions of valid species from 1758 to present.

The accepted starting point of our contemporary system of binomial nomenclature is with the publication of the tenth edition of *Systema Naturae* by Linnaeus in 1758 [8]. In this work, Linnaeus named the genus *Pennatula*, from which the name for the order Pennatulacea is derived. Sixty five percent of the genera and 82% of the species were named between 1858 and 1921, the heyday of new taxon discovery and description. Prior to the 1850’s and after the 1920’s, only modest increases in discovery and description were made (Fig. 5). Eighteen researchers have contributed the 35 valid generic descriptions of pennatulaceans. Kölliker named 24% of the pennatulacean genera, 12% were named by Kükenthal & Broch, Verrill named 9%, as did López-González & Williams. Valenciennes, Tixier-Durivault, Lamarck, and Herklots each named 6%. Lastly, 3% percent of the genera were each named by Cuvier, Hubrecht, Asbjørnsen, Gravier, Studer, Linnaeus, Gray, and Nutting.

For citations to the older references listed in the preceding introduction (between the years 1469 and 1758), see: [8], [15].

### Diversity and Classification

Regarding the extant anthozoan Subclass Octocorallia as a whole, 47 families in approximately 340 genera and over 3200 described species are currently recognized. Included here are the extant Pennatulacea with 14 families, 35 genera; and approximately 450 described species of which an estimated 200 are considered valid (Table 1). These are remarkably similar numbers to stylasterid hydrocorals for species and extant and fossil species (S. Cairns, 1991) [16]. In addition, the fossil pennatulacean taxa include at least 25 species in 7 proposed genera of the Cretaceous and Tertiary Periods [8], [15]. The phylogenetic affinities of the Ediacaran frond-like organisms (the rangeomorphs of 575–560 Ma), although superficially resembling sea pens, have proven highly disputatious and controversial in the literature, and are not considered pennatulacean in nature by a number of paleontologists as well as invertebrate zoologists [17], [18].

**Figure 6:**
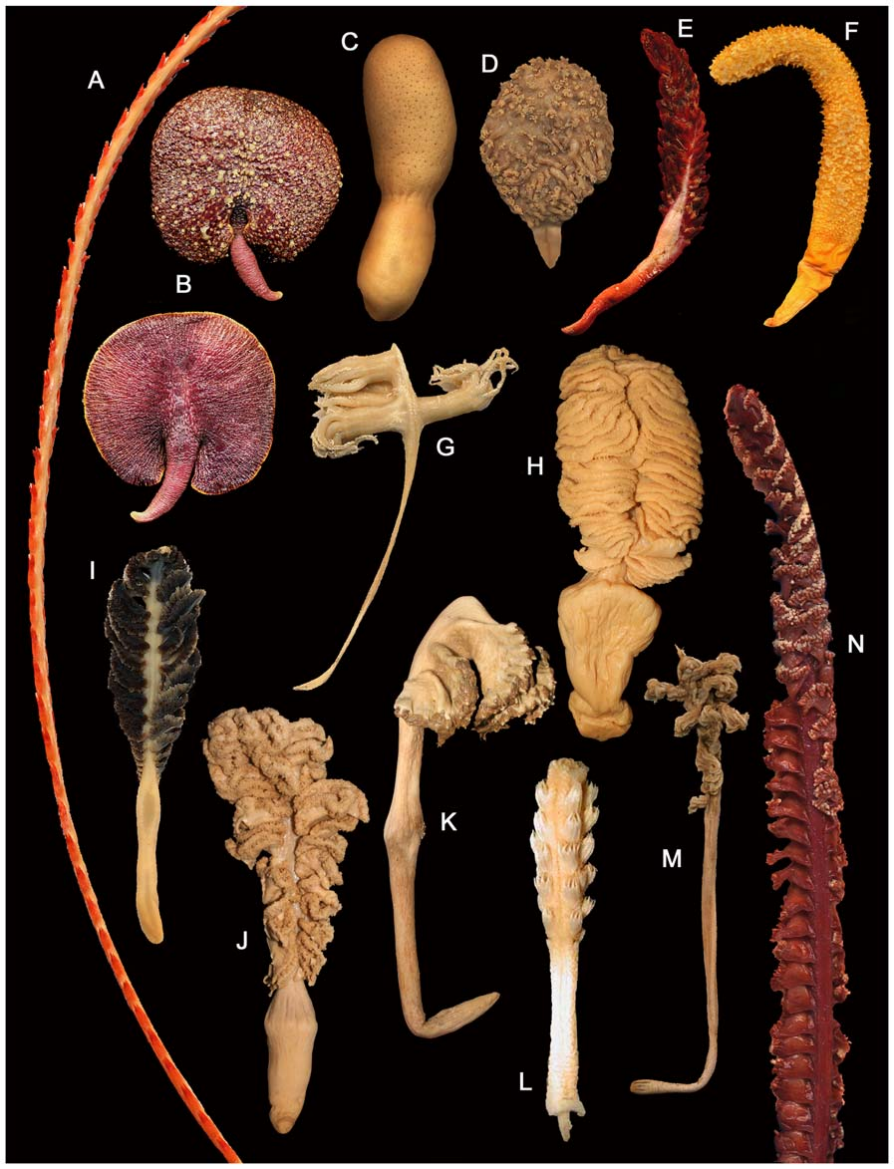
A. *Distichoptilum gracile*, 100 mm. B. *Renilla amesthystina*, 38 mm, dorsal (upper), ventral (lower). C. *Cavernularia glans*, 65 mm. D. *Cavernularia malabarica*, 32 mm. E. *Echinoptilum echinatum*, 57 mm. F. *Actinoptilum molle*, 90 mm. G. *Porcupinella profunda*, 40 mm. H. *Sarcoptilus grandis*, 220 mm. I. *Pteroeides sp.*, 58 mm. J. *Crassophyllum cristatum*, 195 mm. K. *Gyrophyllum sibogae*, 230 mm. L. *Gilibelemnon octodentatum*, 44 mm. M. *Kophobelemnon affine*, 140 mm. N. *Scytalium martensi*, 120 mm. The measurements given represent the total lengths of the entire specimens or portion of specimens shown.

Morphological diversity in the Pennatulacea is remarkable and includes a great variety of growth forms such as plumose, umbellate, clavate, foliate, capitates, digitiform, whip-like, or vermiform (Fig. 6).

The most thorough account of the classification of the Pennatulacea is still that of Kükenthal, published in the early part of the 20^th^ century [19]. Eighty years later, Williams updated classification of the group at the familial and generic levels, and gave a brief account of the nominal and valid species recognized for each genus [7].

### Phylogeny and Evolutionary Biology

Phylogenetic relationships of pennatulaceans must still be considered far from resolved. Neither morphological or molecular phylogenetic data have up-until-now proved adequate to resolve various details of pennatulacean phyogeny. Most taxa have a relative paucity of good morphological characters for use in phylogenetic analysis, and fresh material (especially from great depths) is difficult to acquire. Preserved specimens are often few, difficult to acquire for examination, or poorly preserved for molecular analysis techniques. Relatively few papers have appeared in the past century and half of them have at least partially treated the phylogeny or evolution of pennatulaceans [5], [17]–[33].

Kölliker viewed deep sea taxa as the oldest and most primitive of pennatulaceans and considered the genera *Umbellula* and *Protoptilum* as early derivatives of the pennatulacean prototype [21]. This view can be seen in light of a general popularity of thought at the time that viewed the mysterious abyss as the place of origin of the “primordial ooze” and home to relicts of an ancient marine fauna. Marshall countered Kölliker’s assertion by recognizing the remarkable diversity of deep sea pennatulaceans and derived aspects of species in the genus *Umbellula* [33]. Contrary to this view, Koch considered the veretillids as transitional forms between other octocorals and pennatulaceans [22]. Williams (1994) postulated that pennatulaceans originally diversified in relatively shallow water and subsequently differentiated and dispersed into regions of all depths of the world’s oceans, thus contradicting Kölliker’s view, while at the same time supporting that of Marshall [29]. Many diverse forms inhabit the deep-sea, with derived features, thus the concept of the deep-sea taxa as necessarily plesiomorphic pennatulaceans, is not supported. The recognition of veretillid genera as basal pennatulacean taxa has subsequently been reiterated by Kükenthal, Niedermeyer, Kükenthal and Broch, Hickson, and Williams [17], [24]–[28]. Bourne viewed bilateral symmetry of the rachis and the possession of polyp leaves as derived characters [23]. Bayer recognized the similarity in axial structure between the ellisellid gorgonians and pennatulaceans [5]. More recently, McFadden et al., using molecular data, considered the gorgonian family Ellisellidae and the Pennatulacea as sister groups [31], which consequently would define the gorgonian suborder Calcaxonia as a paraphyletic assemblage [34].

**Figure 7:**
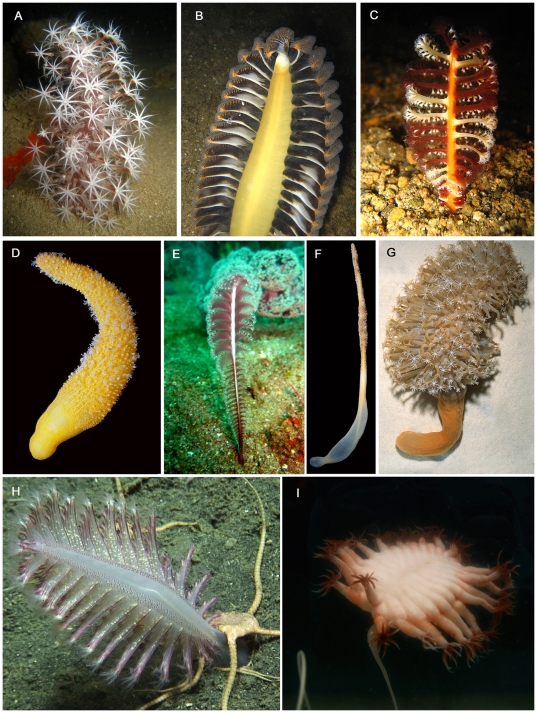
Living pennatulaceans. A. *Veretillum* cf. *manillense*, Philippines; photo courtesy: Terrence Gosliner, California Academy of Sciences. B. *Pteroeides* sp., Philippines. C. *Virgularia* cf. *rumphii*, Philippines. D. *Actinoptilum molle*, South Africa. E. *Scytalium* cf. *sarsi*, Philippines. F. *Sclerobelemnon* sp., Papua New Guinea. G. *Cavernularia* cf. *obesa*, Papua New Guinea. H. *Pennatula phosphorea*, California; photo courtesy: Coral Reef Conservation Program, National Marine Fisheries Service, National Oceanic and Atmospheric Administration. I. *Umbellula* cf. *carpenteri*, South Orkney Islands; photo courtesy: Susanne Lockhart, Antarctic Marine Living Resources Program, Southwest Fisheries Science Center, National Oceanic and Atmospheric Administration.

Evolutionary changes with depth in deep-sea pennatulaceans as contrasted with shallow-water species, include a reduction in the number of the feeding polyps (autozooids) as well as an increase in their size – length and width [11]. Also, pennatulaceans with polyp leaves (Figs. 1C, E and 7B, C, E, H) are found in shallow to middle depths (<3000 m), but are absent in depths >3000 m (Fig. 4). Pasternak describes adaptations and variability of deep-sea pennatulaceans [35].

## Discussion

### Exploration and Discovery

Rapid developments in technology have greatly expanded the frontiers of deep-sea exploration and accessibility, including data collection, photography, and specimen collection. Because of this trend, it is anticipated that discovery and description of pennatulaceans will at least continue if not show a marked increase in the near future.

### Molecular Phylogenetics

The phylogenetics of octocorals based on morphological characters has proven useful in some cases [30], [35], but in general is limited with unclear or unresolved results. Molecular techniques have been expected to help resolve questions of octocoral phylogeny. However, although the expectations or promise of molecular phylogenetics regarding the resolution of phylogenetic problems in the octocorals have resulted in some limited success, overall they have remained largely unfulfilled [32]. A variety of reasons have contributed to this outcome. Firstly, invertebrates such as sponges and coelenterates have been shown to exhibit relatively high levels of genetic variation [36]. More recently, molecular biologists have shown that the resolution necessary to propose alternate classifications for the octocorals based on molecular evidence rather than morphological characters is absent, as is the resolution necessary to effectively illustrate morphological evolution in the group [32]. The slow rate of mitochondrial gene evolution and the absence of molecular markers with sufficient variation to distinguish populations and species (or even genera in some cases) in the Octocorallia have until-now thwarted efforts to do so. Future developments in genomic-sequencing technologies offer the best promise for progress in delineating phylogenetic relationships and character evolution in the octocorals. Some limitations of using molecular techniques in systematics has been addressed for marine fishes [37].

### Recent Studies

Some noteworthy papers treating systematics and the addition of new pennatulacean taxa include the works of at least six authors [38]–[46].
